# Effect of repeating hydrothermal growth processes and rapid thermal annealing on CuO thin film properties

**DOI:** 10.3762/bjnano.15.62

**Published:** 2024-06-24

**Authors:** Monika Ozga, Eunika Zielony, Aleksandra Wierzbicka, Anna Wolska, Marcin Klepka, Marek Godlewski, Bogdan J Kowalski, Bartłomiej S Witkowski

**Affiliations:** 1 Institute of Physics of the Polish Academy of Sciences, Al. Lotnikow 32/46, 02-668, Warsaw, Poland; 2 Department of Experimental Physics, Wroclaw University of Science and Technology, Wybrzeze Wyspiańskiego 27, 50–370 Wroclaw, Poland

**Keywords:** CuO, hydrothermal method, rapid thermal annealing, thin films

## Abstract

This paper presents an investigation into the influence of repeating cycles of hydrothermal growth processes and rapid thermal annealing (HT+RTA) on the properties of CuO thin films. An innovative hydrothermal method ensures homogeneous single-phase films initially. However, their electrical instability and susceptibility to cracking under the influence of temperature have posed a challenge to their utilization in electronic devices. To address this limitation, the HT+RTA procedure has been developed, which effectively eliminated the issue. Comprehensive surface analysis confirmed the procedure’s ability to yield continuous films in which the content of organic compounds responsible for the formation of cracks significantly decreases. Structural analysis underscored the achieved improvements in the crystalline quality of the films. The implementation of the HT+RTA procedure significantly enhances the potential of CuO films for electronic applications. Key findings from Kelvin probe force microscopy analysis demonstrate the possibility of modulating the work function of the material. In addition, scanning capacitance microscopy measurements provided information on the changes in the local carrier concentration with each repetition. These studies indicate the increased usefulness of CuO thin films obtained from the HT+RTA procedure, which expands the possibilities of their applications in electronic devices.

## Introduction

Copper(II) oxide is a p-type semiconductor possessing a narrow bandgap, along with many beneficial electrical, optical, and magnetic properties. Particularly at the nanoscale, these properties set themselves apart and have sparked growing attention on CuO. As a result, CuO has become the focus of both fundamental research and efforts aimed at its practical application across various areas. Nowadays, CuO thin films showing strong hydrophobic properties are tested, among others, in the context of self-cleaning [1], anti-corrosion [2], and antibacterial [3] coatings. Like other CuO nanostructures, thin films also show potential for applications in photovoltaic cells [4–5], lithium-ion batteries [6], supercapacitors [7], gas sensors [8], and biosensors [9]. Furthermore, the literature reports their utility in photodetectors [10–11], memory structures [12–13], and p-type channels in transistors [14–15].

Simultaneously, ongoing efforts are consistently directed towards developing deposition techniques and functionalization of CuO films in order to modify their properties for specific applications. Despite the existence of numerous methods for thin film growth based on various physical and chemical phenomena, it remains acknowledged that these techniques do not yield ideal outcomes in terms of applications in any area [16]. Therefore, comprehending the potential and limitations of these different methods becomes important for their continued enhancement and optimization. Such endeavors might consequently lead to the development of novel techniques for producing films tailored to specific parameters and purposes. The preparation of CuO thin films using various techniques is reported in the literature. These encompass methods such as molecular beam epitaxy [17–19], direct current magnetron sputtering [4,20–21], and pulsed laser deposition [22–24]. Alternative approaches involve techniques such as chemical vapor deposition [25–27] and atomic layer deposition [28–30]. CuO thin films can also be grown from liquid phases, employing techniques such as chemical bath deposition [31–33], successive ionic layer adsorption and reaction (SILAR) [34–36], sol–gel processes (using dip and spin coating) [37–39], as well as spray pyrolysis [40–42]. This work focuses on hydrothermally grown CuO films. There are only a few reports in the literature indicating the use of this method for the growth of CuO films. An example of a multistep method of producing CuO films has been described in [43]. It combines the hydrothermal process, annealing, and spin coating. Conversely, direct thin film growth through the hydrothermal approach is outlined in [44–45]. In a different study [46], CuO films were grown on glass substrates coated with CuHNO_4_ through 4, 8, and 12 h long hydrothermal treatments of copper hydroxide nitrate suspension at 200 °C. Another example is the work [47], outlining the fabrication of structured CuO films on fluorine-doped tin oxide from a solution devoid of additional substances.

Post-processing plays a significant role in the field of nanomaterials fabrication. Metal oxide thin films often undergo annealing. This practice aims to improve the properties of these layers and to attain the desired parameters in the final product. Controlled thermal treatment serves various purposes, such as enabling crystal structure relaxation, defect reduction, and enhancement of the films’ crystalline arrangement. Furthermore, it can lead to improved electrical conductivity and changes in optical attributes.

The CuO films that are the subject of this work were grown using a novel approach to the hydrothermal method. They are produced from aqueous copper(II) acetate solutions heated to approximately 95 °C. Notably, a key distinction of these growth processes is their execution in an open system, accomplishing synthesis within significantly shorter durations (from 48 s up to 6 min) than those reported in the aforementioned literature [48]. Mild growth conditions enhance the appeal of the technology by fostering energy efficiency, cost-effectiveness, and safety. It aligns with the concept of rapid hydrothermal reactions, which is one of the trends in the modern advancement of the method. The work carried out in this area is aimed at obtaining specific materials in the shortest possible time. Taking into account the limited electrical stability of the so-prepared CuO films, their properties were modified by thermal post-processing. The resultant procedure of sequential hydrothermal processes and rapid thermal annealing (HT+RTA) allows for the control of the physical properties of CuO films.

## Experimental

### Sample preparation

The analyzed films were fabricated on n-type silicon (100) substrates (Siegert Wafer). These substrates were covered with a nucleation layer in the form of gold nanoislands sputtered using a turbomolecular-pumped coater Quorum Q150T ES. CuO films were obtained according to the following procedure. First, an aqueous solution of copper(II) acetate (Chempur) with a concentration of Cu(II) ions of 1 mM was prepared. The mixture was then precipitated by adding NaOH until a pH of 6.5 was attained. Subsequently, the so-prepared solution along with the substrate was placed in a reaction vessel and uniformly heated utilizing an induction cooker (heating power = 3.5 kW) up to a boiling temperature of ca. 95 °C and boiled for 30 s. After this, the sample was removed from the vessel and cleaned in an ultrasonic cleaner in isopropanol for 30 s and subsequently dried with nitrogen. The resultant as-grown film underwent rapid thermal annealing (RTA, AccuThermo AW610, Allwin21) at 450 °C for 5 min. The heating rate was 45 °C/s. This thermal processing took place in an O_2_/N_2_ atmosphere at a 1:1 ratio. The complete sequence of the hydrothermal growth process and rapid thermal annealing steps is referred to as the HT+RTA cycle. Samples that underwent this procedure are denoted as “1×”, “2×”, and “3×”, depending on the number of HT+RTA cycles. It is worth highlighting that re-executing the hydrothermal processes does not necessitate any supplementary preparation of samples, for example, surface re-nucleation.

### Measurement equipment

CuO thin films were characterized using an analytical scanning electron microscope (SEM) Hitachi SU-70 with the X-ray microanalysis system (EDX) enabling composition analysis (Thermo Fisher Pathfinder system with a silicon drift detector). Additionally, in the study, a scanning probe microscope (Dimension Icon, Bruker) was used, which allowed for the investigation of both topography and electrical properties of the films. Surface topography analysis was performed by utilizing an atomic force microscopy (AFM) operating in Peak Force Tapping mode. The surface was scanned at a resolution of 1024 × 1024 measurement points using a silicon nitride probe, ScanAsyst-AIR (Bruker). Scanning capacitance microscopy (SCM) measurements were conducted in contact mode using a silicon probe coated with a PtIr layer, SCM-PIT-V2 (Bruker). Capacitance measurements were taken with *V*_AC_ = 2 V and *V*_DC_ = 1 V applied. The carrier distribution maps at a resolution of 256 × 256 pixels presented in the paper were derived from the “SCM data” channel. Contact potential difference (*V*_CPD_) measurements were carried out using Kelvin probe force microscopy (KPFM) in amplitude modulation mode, also employing SCM-PIT-V2 probes from Bruker. These measurements were performed under ambient conditions, which may have introduced surface states or absorbed water molecules on the surface of the investigated samples. Consequently, the obtained *V*_CPD_ values and the resulting work function values are susceptible to additional errors, including those arising from the screening effect [49–50]. Despite the measurement conditions, it was still possible to qualitatively analyze changes in surface potential and work function based on the sample preparation method. The AFM, SCM, and KPFM data were analyzed using the Nanoscope Analysis 3.0 software (Bruker). The CuO films also underwent structural analysis using a high-resolution X-ray diffractometer X’Pert Pro MRD (Panalytical) equipped with a Cu anode (λ = 1.54060 Å).

X-ray photoelectron spectroscopy (XPS) measurements were conducted utilizing a Scienta R4000 hemispherical analyzer with a pass energy of 200 eV and monochromatic Al Kα (1486.7 eV) excitation (Scienta MX-650) operating at 150 W. The energy scale was calibrated by setting the C–C bond at 285 eV. Analysis of the spectra was performed using the commercial CASA XPS software package (Casa Software Ltd., version 2.3.17) with Shirley background. The Gaussian–Lorentzian functional GL(30) was applied during the simulations. Raman scattering measurements were performed under ambient conditions and room temperature using a T64000 Horiba Jobin-Yvon spectrometer configured in a backscattering geometry with a 1800 gr/mm grating and a microscope objective of 100× magnification. The spectral resolution was of the order of 0.5 cm^−1^. A 532 nm semiconductor laser was used to illuminate the samples. The measurements were performed without detection of polarization of the scattered light. A liquid nitrogen-cooled multichannel silicon CCD camera was used as a detector.

## Results and Discussion

**Figure 1:**
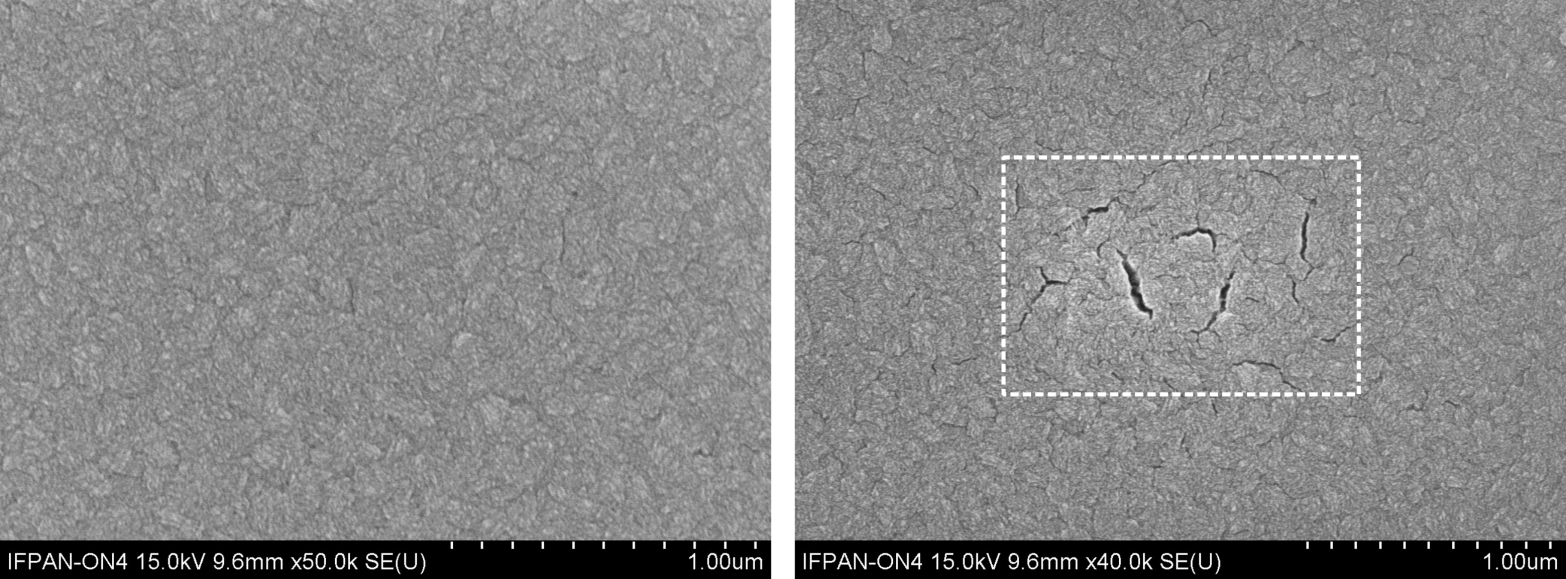
SEM images of the films surface prior to (left) and after exposure of the indicated area to an electron beam for 30 s (right).

The as-grown films are uniform and consist of single-phase CuO. However, they exhibit a notably high content of organic compounds, which are residues from the growth process. These residues contribute to the films’ low electrical stability and their susceptibility to temperature-induced cracking. This phenomenon was manifested, for example, during SEM measurements, when the sample was locally heated by an electron beam, as shown in Figure 1.

Simultaneously, exposing the samples to thermal processing results in a reduction of the content of carbon atoms. On this basis, it is postulated that, under the influence of temperature, organic compounds are released from the material, leading to the formation of discontinuities within the layers. Therefore, the HT+RTA sequencing procedure was developed.

The modification aimed to obtain continuous layers with minimal carbon compound content, reducing cracking under the influence of temperature. Additionally, it was crucial to prevent the formation of foreign crystallographic phases of copper compounds, such as Cu_2_O. To achieve this, careful optimization of the procedure was undertaken, involving the examination of nearly 450 combinations of the parameters. These factors included single film thickness, substrate type and atmosphere, temperature, and time of annealing along with the number of repetitions of HT+RTA cycles, which is the focus of this study. The most favorable results, that is, layers with the highest level of continuity and reduced carbon content, were achieved by annealing samples at 450 °C for 5 min in O_2_/N_2_ (1:1) atmosphere. Table 1 provides a summary of the average thicknesses and elemental content of the CuO films. Notably, EDX results demonstrate that the newly developed HT+RTA approach significantly reduces the carbon content in the CuO films. The chemical composition of such thin films determined using EDX is only an approximation. However, the obtained results demonstrate a clear trend of decreasing carbon content in the material with successive HT+RTA cycles.

**Table 1:** Summary of the average thicknesses of the analyzed samples and their elemental composition.

Sample	Average thickness (nm)	Elemental composition (atom %)		

		O	Cu	C

as grown	70	50.5 ± 0.3	42.7 ± 0.6	6.9 ± 0.3
1×	68	45.9 ± 0.8	50.9 ± 0.6	3.2 ± 0.3
2×	136	46.6 ± 0.6	51.5 ± 0.5	2 ± 0.2
3×	213	44.8 ± 0.5	53.6 ± 0.4	1.6 ± 0.2

**Figure 2:**
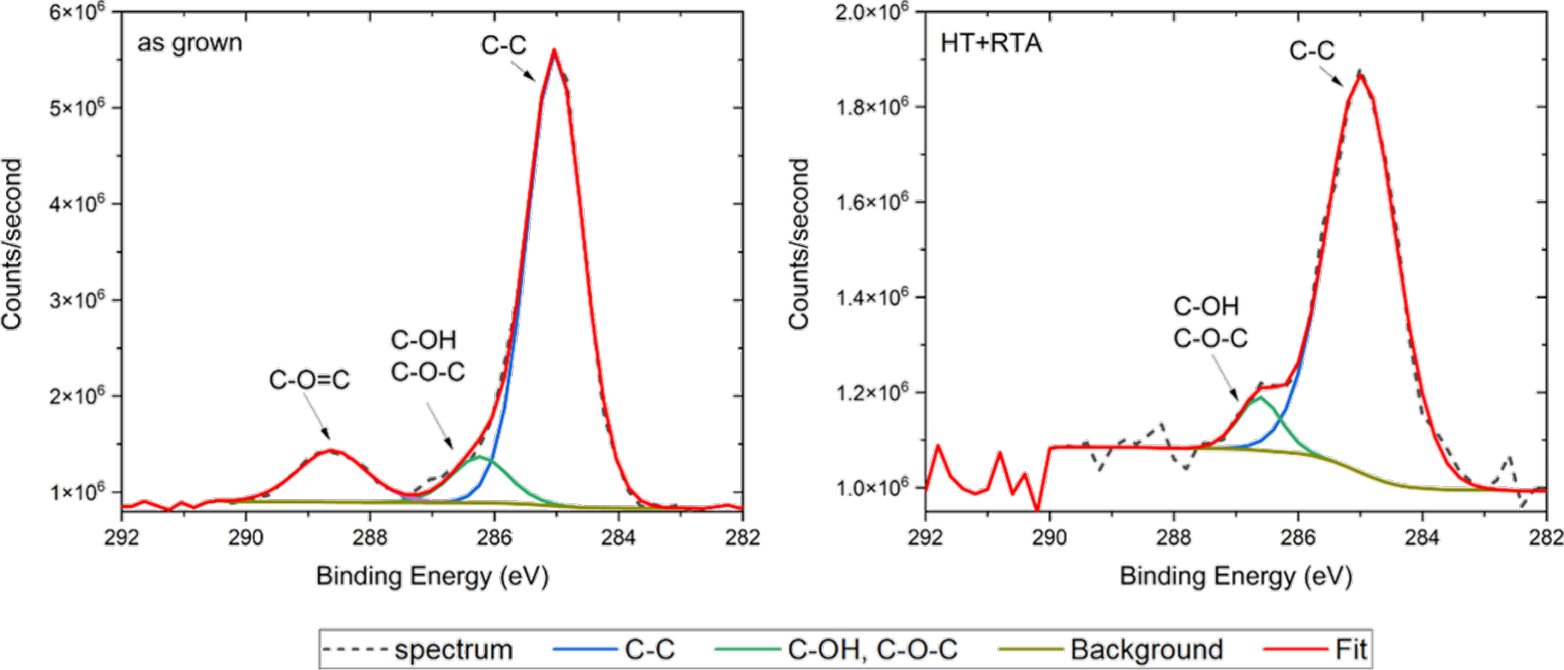
The C 1s peaks in high-resolution XPS spectra of as-grown films (left) and processed using the HT+RTA procedure (2×) (right).

To validate those findings, additional XPS analyses were conducted on the as-grown and 2× samples. The results (Figure 2) obtained for the C 1s band reveal a higher carbon content in the as-grown sample compared to the 2× sample. In both samples, a signal corresponding to C–C bonds at 285 eV and C–OH and C–O–C bonds at around 286.5 eV was observed [51]. Notably, the O–C=O bond peak at 288.6 eV was exclusively detected in the as-grown films, proving that the HT+RTA procedure leads to the release of carboxyl groups originating from the copper(II) acetate precursor.

**Figure 3:**
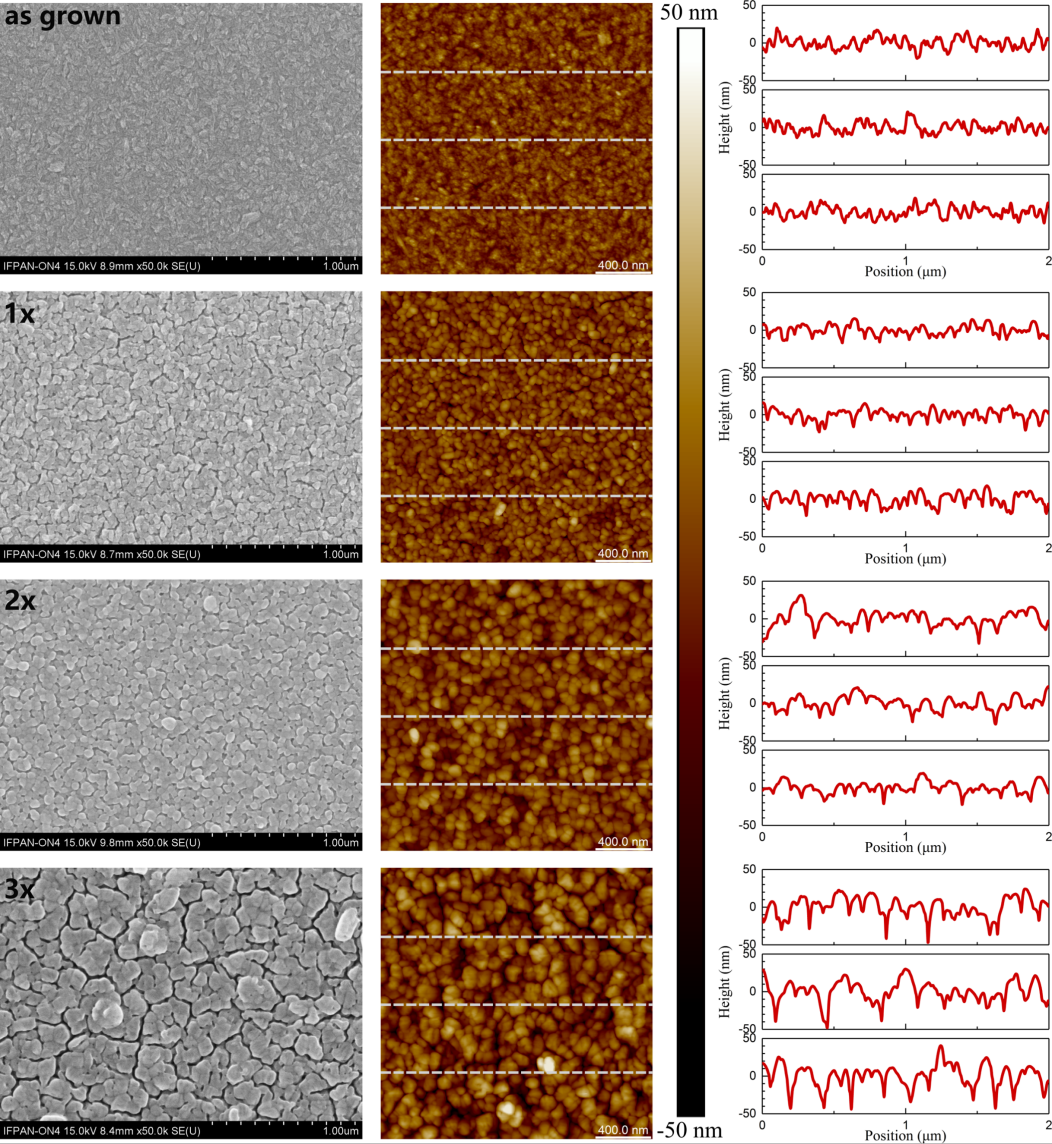
SEM and AFM images of the surface of the as-grown, 1×, 2×, and 3× samples, as well as topography profiles along the marked lines (every 500 nm) presented at the same scale.

A comprehensive surface analysis of the as-grown films and those prepared by repeating the HT+RTA cycle one, two, and three times was conducted using SEM and AFM. Figure 3 shows representative images of the surface and height profiles (where 0 corresponds to the mean plane) acquired along the marked lines from the AFM scans of the analyzed samples.

The measured parameters are compiled in Table 2. The surface of the as-grown films is characterized by an even, random arrangement of grains, elongated in a single direction, with a size of approximately 50 nm. These grains consist of agglomerates of nanocrystallites. However, in the case of sequenced films (after the HT+RTA procedure), those nanocrystallites are not as clearly distinguishable. Under the influence of temperature, atoms can diffuse along the boundaries between particular crystallites, which leads to their coalescence. Moreover, annealing of polycrystalline films can lead to grain boundary migration to reduce the surface energy of the system [52]. Both of these mechanisms contribute to an increase in grain size with each successive repetition of the HT+RTA cycle. Another consequence of thermal processing is a change in the surface structure. SEM images reveal that the number of sequencing cycles affects the continuity of the films. When analyzing the height profiles, it is observed that in the 1× samples, the height differences are comparable to the average thickness of the film. It suggests that some of the visible discontinuities extend all the way to the substrate. In contrast, for triply sequenced films, although the differences in height are smaller relative to the average thickness of the material, the gaps between agglomerates of grains are deep (reaching up to 80–90 nm) and wide, which makes the samples unsuitable for electrical measurements. The most favorable outcomes were observed for 2× samples, as only a few discontinuities are observed on the surface, which are narrow and shallow compared to the average thickness of the films.

**Table 2:** The analyzed parameters of the surface of the as-grown and sequenced films determined from AFM measurements.

Sample	*R*_q_ (nm)	*R*_a_ (nm)	*R*_q_/*R*_a_	*R*_sk_	*R*_ku_

as-grown	5.84	4.66	1.253	0.182	3.13
1×	7.46	5.94	1.256	0.386	3.81
2×	9.31	7.45	1.249	0.081	3.29
3×	15.1	11.75	1.285	−0.313	3.68

Based on the surface parameters determined from AFM data, that is, root mean square (*R*_q_) and arithmetic average (*R*_a_) of height deviations, it was noticed that the surface roughness progressively increases with each HT+RTA cycle. The most significant increase was observed in films that underwent three sequential cycles of HT+RTA, primarily because of the presence of numerous discontinuities. Furthermore, the HT+RTA sequencing results in a variation of height distributions, as indicated by the skewness (*R*_sk_) and kurtosis (*R*_ku_). The most homogeneous surface coverage is observed for the as-grown and 2× samples. However, the 3× sample is the only one characterized by negative skewness, which, together with the high value of kurtosis, indicates that the surface is slightly dominated by steep valleys. The *R*_q_/*R*_a_ ratio of approx. 1.25 implies that, despite those discontinuities, the height distributions do not show large deviations from the normal distribution [53]. In conclusion, the 2× sample exhibits the most favorable surface parameters among all samples treated with the HT+RTA procedure. It demonstrates homogeneity without a notable dominance of hills or valleys.

**Figure 4:**
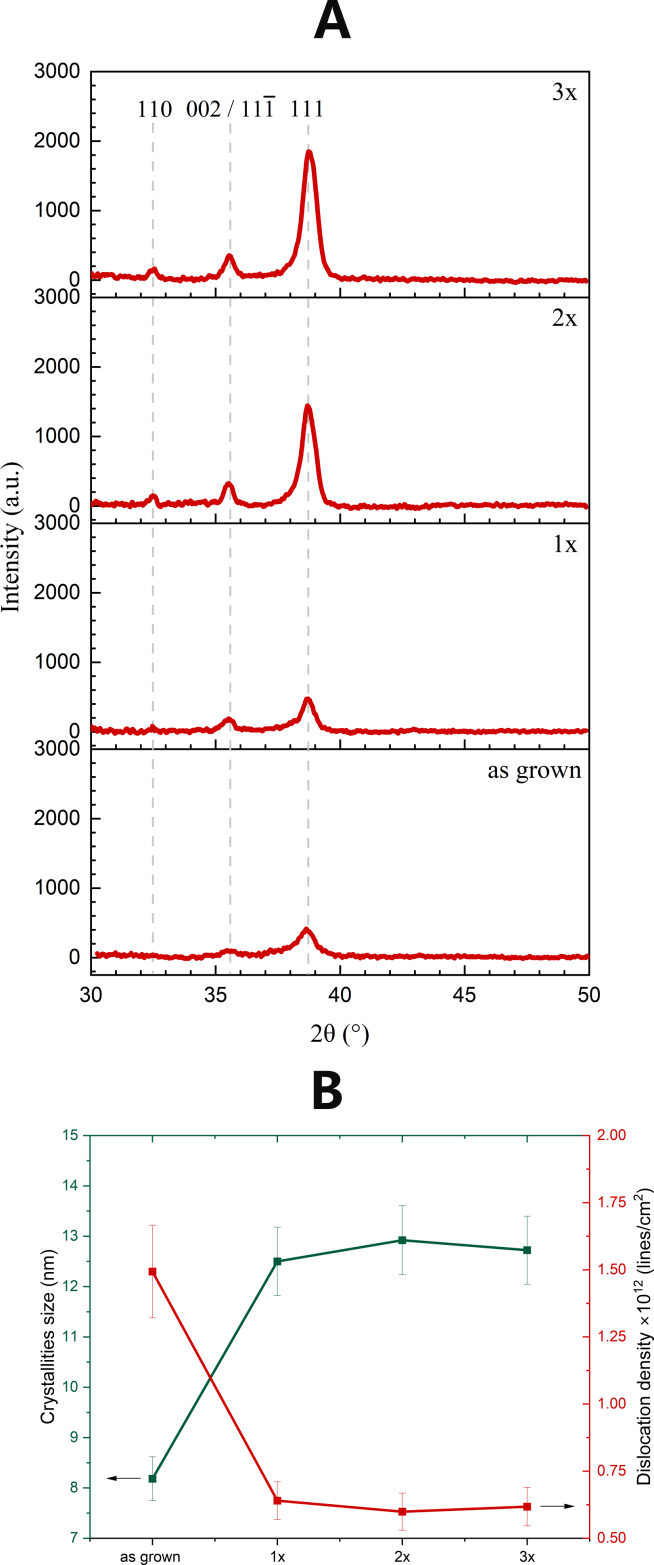
(A) Diffractograms and (B) variations in crystallite sizes and dislocation density determined for as-grown and HT+RTA films.

The structural properties of the CuO films were evaluated using XRD and Raman spectroscopy. The XRD diffractograms (Figure 4A) exhibit well-defined reflections that correspond to the polycrystalline monoclinic structure of CuO (JCPDS card no. 00-048-1548). Notably, no peaks of foreign crystallographic phases (such as Cu_2_O) were recorded regardless of the sample preparation. This can be attributed to the appropriate selection of annealing parameters, including temperature and the ratio of O_2_ to N_2_. Regarding the as-grown films, only three reflections located at 2θ ≈ 35.57° (002)/(11−1) and 2θ ≈ 38.66° (111) were observed. In contrast, the diffractograms of the sequenced films (1×, 2×, and 3×) contain an additional peak at 2θ ≈ 32.5° (110), whose intensity increases with each HT+RTA cycle. The appearance of an additional peak indicates an improvement in the crystallographic quality of these layers. Regardless of the examined films, the preferred (111) orientation remains consistent. Analysis of its position shows a slight shift towards higher angles observed with each subsequent repetition of the HT+RTA procedure. Analysis of the predominant peak was conducted to assess variations in crystallite size and dislocations (Figure 4B). A distinct contrast between the as-grown and 1× sample is observed for each of these parameters, whereas the graph flattens for the sequenced samples. The nanocrystallite size was estimated using the Scherrer equation [54–55] with sizes ranging from 8.18 nm for the as-grown samples to 12.50, 12.92, and 12.72 nm for the 1×, 2×, and 3× samples, respectively. The other parameters demonstrate an opposite trend, showing a decrease compared to the as-grown films. Reduction of full width at half maximum (FWHM) as well as dislocation density values after annealing the samples at 450 °C indicates a reduction in structural defects and an enhancement in the crystallographic quality of the produced material. The determined parameter values for the 1×, 2×, and 3× samples exhibit only a minor range of variation. On this basis, it is concluded that the number of HT+RTA cycles has a minimal impact on the structural properties of the prepared CuO films. This result further validates the high reproducibility of the developed sequencing procedure.

Three different copper oxide phases can be obtained, namely, cupric CuO, cuprous Cu_2_O and the intermediate phase paramelaconite Cu_4_O_3_. The aforementioned phases of copper oxide have different physical and electrical properties, different colors, and crystal structures [55]. By examining the Raman spectra of copper oxide compounds, phase as well as chemical composition can be identified. Each form of copper oxide presents a different Raman spectrum.

**Figure 5:**
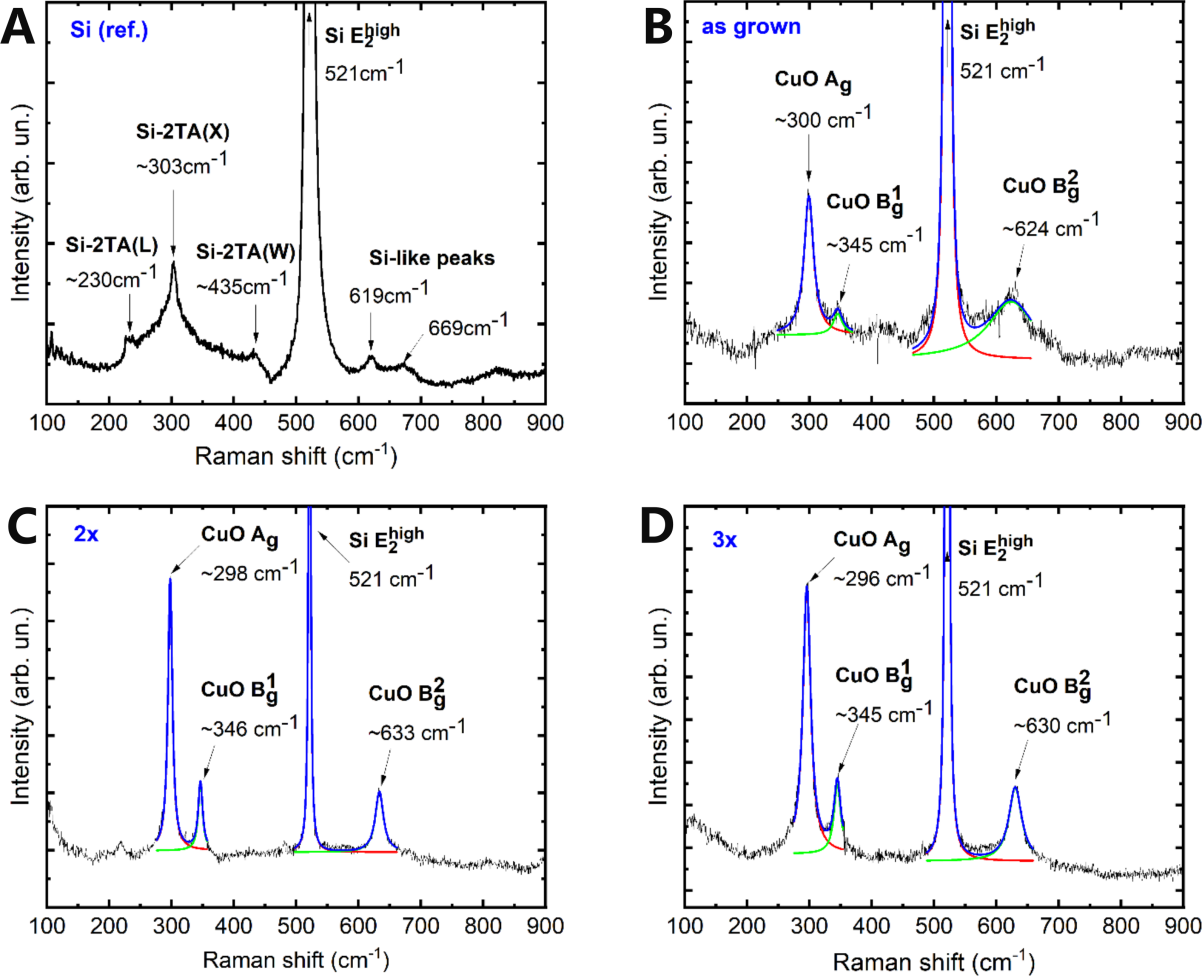
Raman spectra of as-grown (B), 2× (C), and 3× (D) CuO/Si structures along with that of a reference Si sample (A) for comparison.

CuO crystallizes in a monoclinic lattice with the space group 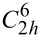 giving the following set of the zone-center lattice modes: Γ = *A_g_* + 2*B_g_* + 4*A_u_* + 5*B_u_*. Hence, CuO has twelve phonon branches in the phonon dispersion spectrum as there are four atoms in the primitive cell. Out of these modes, only the *A_g_* and two *B_g_* modes are Raman-active. The next three modes (*A_u_* + 2*B_u_*) are acoustic, whereas six modes (3*A_u_* + 3*B_u_*) are IR-active [56–57]. Figure 5 shows the Raman spectra measured for as-grown, 2× and 3× CuO/Si samples. A reference sample (Si ref.), which was a pure silicon wafer, was also studied for comparison (Figure 5A).

Comparing the Raman spectra of CuO/Si samples with the spectrum of the Si reference sample, one can notice a dominating 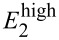 phonon mode at a frequency of 521 cm^−1^ originating from the silicon substrate [57]. The remaining Raman lines, which were fitted with Lorentz functions, reveal mode frequencies in the ranges of 296–300, 345–346, and 624–633 cm^−1^. These values correspond to the following vibrations in the CuO layer: CuO *A_g_* ≈ 300 cm^−1^, CuO 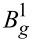 ≈ 345 cm^−1^, and CuO 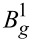 ≈ 630 cm^−1^. Their values are similar to the CuO-like phonon mode frequencies that have been documented by other authors [56,58–60]. Thus, the obtained results confirm the cupric phase of the copper oxide layer. After comparing the Raman spectra of all CuO/Si structures, it can be noticed that the 2× and 3× samples exhibit the best crystal quality among the studied structures because they have well-resolved and the most exposed CuO-like *A_g_*, 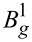, and 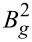 phonon modes in their Raman spectra.

The surface potential (i.e., contact potential difference, *V*_CPD_) and work function (ϕ) variations resulting from the HT+RTA sequencing were examined using KPFM. First, following the procedure described in [61], the work function of the probe was determined (ϕ_tip_ ≈ 4.95 eV), which is consistent with the literature [62–64]. Then, the relation ϕ_CuO_ = ϕ_tip_ − *eV*_CPD_ was used to calculate the work function of the CuO films.

The work function represents the sum of chemical potential and surface dipoles, which are sensitive to numerous factors. In addition to the stoichiometry of the material and the presence of impurities, their value may also be affected by, among others, surface roughness, exposed crystal plane, and exposure to radiation or gasses that may be absorbed at the surface [65]. The measured *V*_CPD_ may considerably deviate from the real work function values as some of these factors influence the surface studied under ambient conditions. Nevertheless, because constant conditions were maintained for each of the analyzed samples, it is possible to determine the general trend of parameter changes with subsequent HT+RTA cycles.

**Figure 6:**
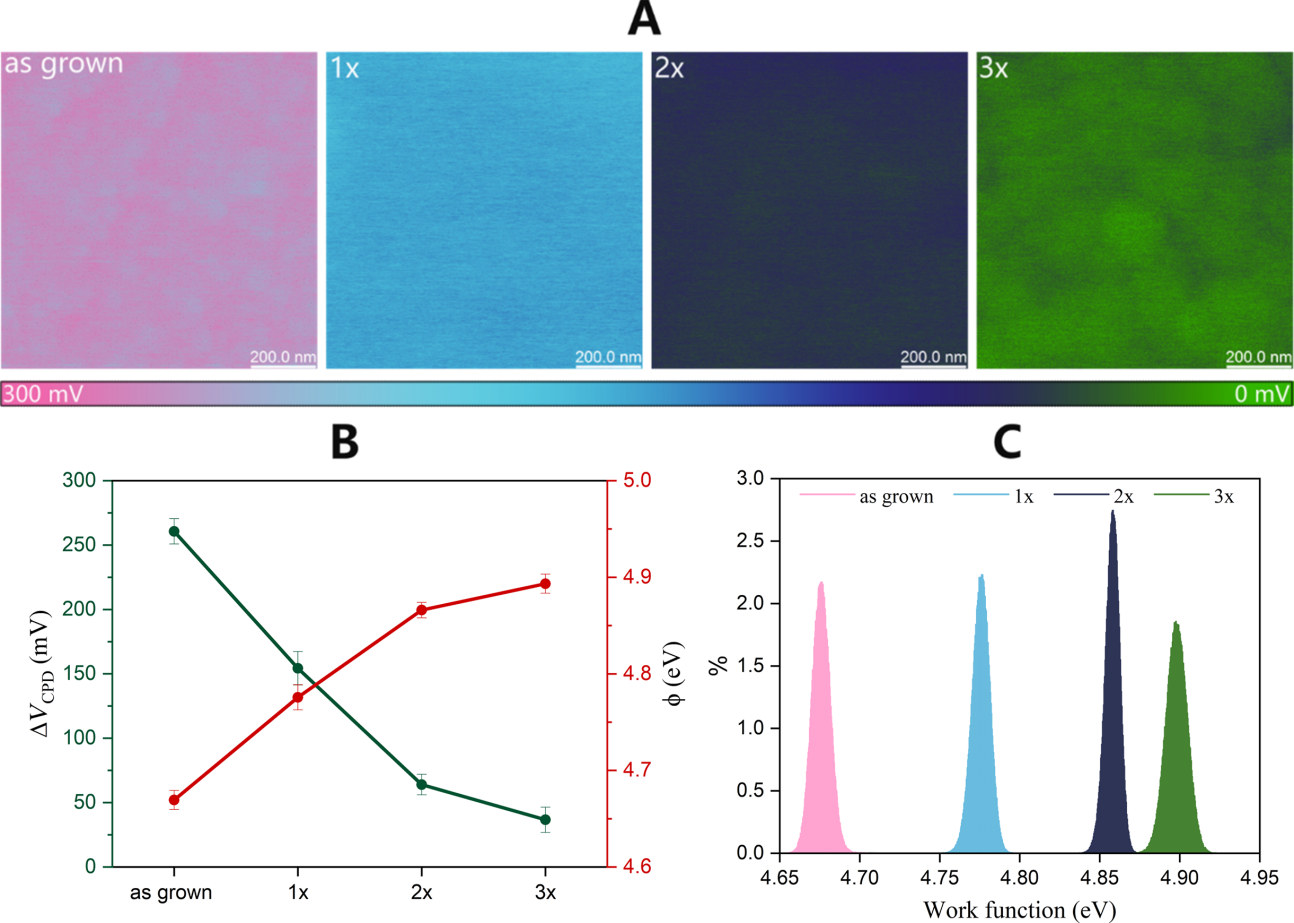
(A) Exemplary maps (1 × 1 μm^2^) of the contact potential difference (*V*_CPD_) for each sample. (B) Summary of the average values of *V*_CPD_ and ϕ. (C) Distributions of the work function values.

Figure 6A presents exemplary *V*_CPD_ maps for all samples, which show a wide range of *V*_CPD_ values. The average values of *V*_CPD_ determined through measurements at three points of each sample are depicted in Figure 6B. The average values range from 260 mV in the case of as-grown films and then decrease with each subsequent HT+RTA cycle, reaching the lowest value of 38 mV for the 3× sample. As a consequence, the average values of ϕ vary from 4.66 eV for as-grown films to 4.89 eV for the 3× sample. Several factors can cause the work function to increase with successive HT+RTA cycles. Undoubtedly, the annealing of the samples greatly affects the properties of the material. As shown earlier, a number of changes occurs as a result of annealing. The most important of them in the context of the work function are a reduction of defects, contamination of the film, as well as reconstruction of the film surface. Important information is provided by the histograms of the work function values as their shape indicates the non-uniformity of the contact voltage in the analyzed area. On the presented maps, a slight difference in the *V*_CPD_ values (varying by several millivolts) between the grain boundaries and their surface can be observed. Analyzing the ϕ distributions, it can be inferred that the 2× layer, exhibiting the lowest FWHM, demonstrates the highest homogeneity in potential distribution. This finding further supports the argument that repeating the HT+RTA cycle twice is optimal.

The change in contact potential difference provides valuable information about the direction and degree of band bending resulting from surface states. [50] The previously discussed results do not indicate significant differences in the structural properties and elemental composition among the 1×, 2×, and 3× samples. Hence, it is believed that the most probable cause for the increasing work function value is the annealing atmosphere of the samples (O_2_/N_2_, 1:1) [66]. The presence of oxygen in the annealing atmosphere fills the oxygen vacancies present in the material, consequently leading to an elevation in the work function value. The combination of O_2_ and N_2_ was chosen over pure oxygen because of cost-effectiveness. At the same time, efforts were made to ensure that the selected annealing atmosphere was closer to the composition of air. Nevertheless, additional investigation is required to validate this hypothesis.

**Figure 7:**
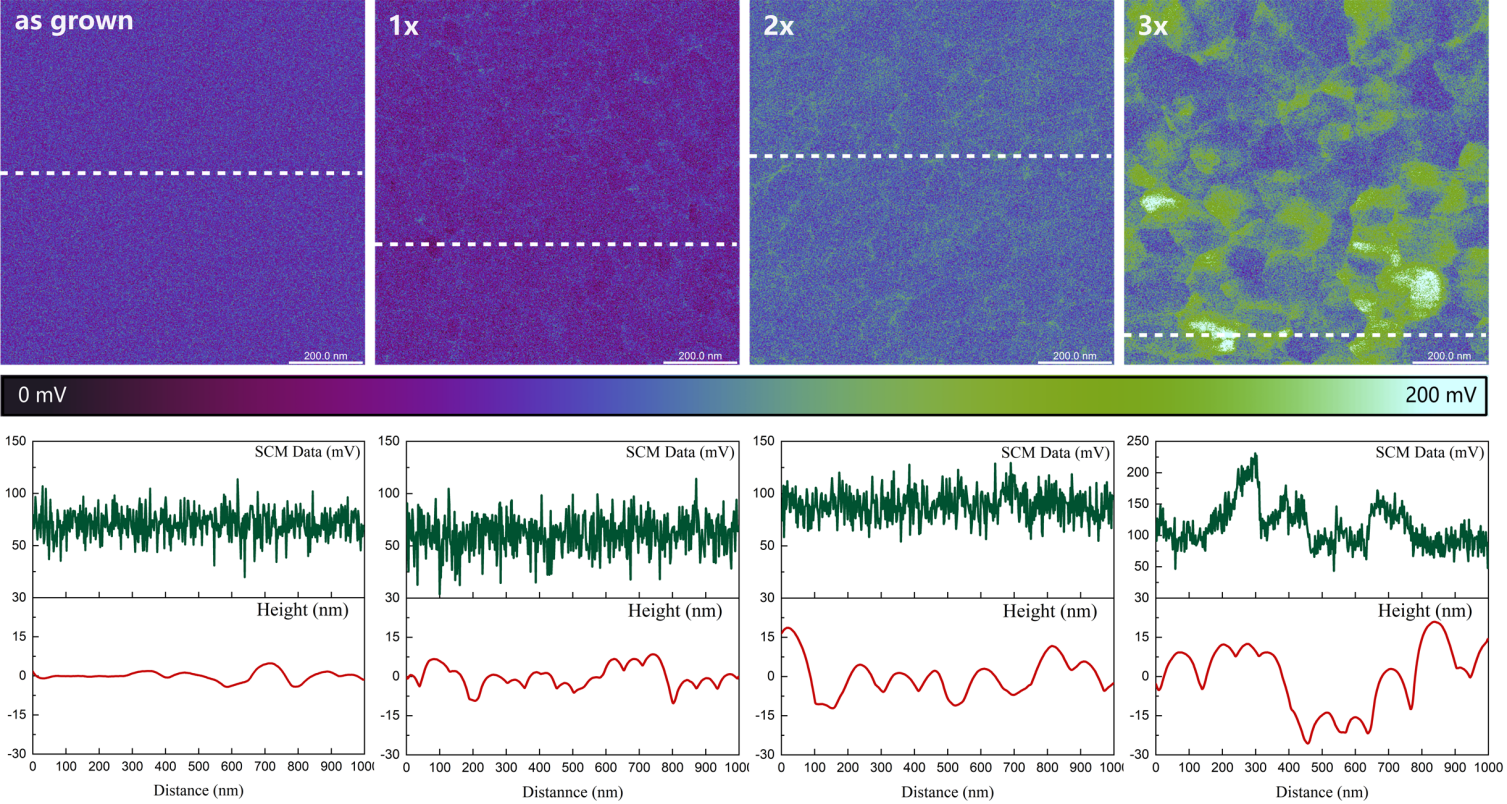
The d*C*/d*V* signal maps of the analyzed samples with the SCM data and height profiles recorded along the marked lines.

Considering the variations in the contact potential at grain boundaries observed during KPFM measurements, the samples additionally underwent SCM investigations to evaluate the distribution of charge carriers on the tested surfaces. The results of this analysis are presented in Figure 7. Regardless of the analyzed sample, the d*C*/d*V* signal consistently exhibits positive values, indicating that there is no carrier inversion due to HT+RTA sequencing. Regardless of the sample preparation procedure, the p-type conductivity prevails in the hydrothermally grown CuO. By examining the root mean square of the SCM signal, it can be observed that annealing the as-grown sample slightly increases the carrier concentration within the studied material. However, this parameter subsequently decreases with each subsequent repetition of the process. Considering the presented maps of contact potential difference and SCM data along with the profiles of the SCM signal and height along the marked lines, it can be seen that the developed sequencing procedure has a large impact on the distribution of charge carriers. In the case of as-grown films, the distribution of carriers is homogeneous and does not show any correlation with the surface topography. The results obtained for the sequenced samples are different. With each successive cycle there is an increasing differentiation of the values of capacitance changes. In the case of 1× and 2× samples, only small, local changes in the SCM signal are observed along the boundaries of some grains. Higher d*C*/d*V* values indicate that the concentration of carriers in these places is lower than on the surface of the grains. The film after a single annealing also shows a few areas with a lower signal, which means that the electrical properties of some grains had changed. These inhomogeneities may be caused by various factors. One is the effects occurring at grain boundaries or structural defects in the boundaries. The defects disrupt the crystalline order and affect the electronic structure near the grain boundaries. Because of their electronic structure grain boundaries can also trap carriers. These phenomena may account for the changes in the d*C*/d*V* signal observed at the corresponding locations. The greatest variation of the d*C*/d*V* signal was recorded for the 3× sample. Analyzing the corresponding profiles, it can be seen that differences in the value of potential changes occur not only at grain boundaries but also between individual grains. This suggests that, as a result of repeating the HT+RTA cycle three times, the carriers are depleted within a large part of the grains, which disturbs the current flow. The most probable cause of such clear discrepancies may be a heterogeneous change in the chemical composition or distribution of defects as a result of thermal treatment. Capacitance differences between individual grains, which are observed to a small extent in the 1× and 2× samples, and their amplification in the case of a triply repeated HT+RTA sequence, suggest that too many cycles can lead to the formation of inhomogeneities in the chemical composition of the manufactured material.

## Conclusion

The search for new growth methods of nanomaterials and the continuous enhancement of existing techniques hold significant importance, particularly within the realm of producing materials for specific applications. CuO thin films produced through hydrothermal processes were initially targeted for electronic applications. However, the low electrical stability of the as-grown films, attributed to the presence of organic residues within the samples, necessitated the development of innovative solutions. These solutions aimed to lower the organic compound content while concurrently enabling modifications to the resultant material’s properties.

As elucidated in this study, the sequential execution of hydrothermal growth processes followed by rapid thermal annealing has a positive influence on material characteristics. Analysis of the films’ elemental composition reveals that repeating HT+RTA cycles leads to a significant reduction in carbon compound content within the produced CuO thin films. Consequently, it has a favorable impact on both electrical and thermal stability. Moreover, as evidenced by XRD and Raman spectroscopy analyses, the sequencing enhances the crystal quality of the films, which remain free from other copper compound phases regardless of the preparation procedure. The absence of notable changes in crystallographic structure also implies that the organic compounds present in the films after hydrothermal processes do not integrate into the CuO crystal lattice. Rather, they become entrapped between grains during the dynamic film formation process. This phenomenon likely accounts for their subsequent release from the layers at relatively moderate temperatures, resulting in the formation of discontinuities along the grain boundaries.

Furthermore, an interesting observation pertains to alterations in the surface potential of the films, directly impacting their work function. This modulation of work function holds immense significance, particularly in the context of electronic applications. Subsequent investigations will delve into comprehending the mechanism underlying the change in the work function value. Particularly, the impact of oxygen concentration in the annealing atmosphere on this parameter will be studied in the future. Another noteworthy observation is the change in the carrier distribution, as depicted by SCM. While the singly and doubly sequenced films exhibit minor changes, the 3× sample exhibits distinct regions of reduced carrier concentration at grain boundaries and surfaces of individual grains. Further research is required to gain insight into the phenomena at grain boundaries, aiming to provide a precise explanation of the factors contributing to such a rapid change in the distribution of carriers in CuO films.

## Data Availability

The data that supports the findings of this study is available from the corresponding author upon reasonable request.
